# Total Laryngectomy—Still Cutting-Edge?

**DOI:** 10.3390/cancers13061405

**Published:** 2021-03-19

**Authors:** Thomas K. Hoffmann

**Affiliations:** Department of Otorhinolaryngology, Head and Neck Surgery, Ulm University Hospital, 89070 Ulm, Germany; t.hoffmann@uniklinik-ulm.de; Tel.: +49-731-500-59501; Fax: +49-731-500-59502

**Keywords:** total laryngectomy, carcinoma, tracheostomy, voice, radiochemotherapy

## Abstract

**Simple Summary:**

Complete removal of the larynx (total laryngectomy) offers a curative approach for advanced laryngeal and pharyngeal cancer. If the operation is performed after radiotherapy wound healing problems have to be taken into account which can be managed by adapted reconstructive techniques. Laryngectomy results in the loss of voice which can be managed e.g., by using a voice prosthesis with a significant increase in quality of life. Total laryngectomy still represents a relevant surgical procedure in modern head and neck oncology.

**Abstract:**

Surgical removal of the larynx (total laryngectomy) offers a curative approach to patients with advanced laryngeal and hypopharyngeal (squamous cell) cancer without distant metastases. Particularly in T4a carcinoma, laryngectomy seems prognostically superior to primary radio(chemo)therapy. Further relevant indications for laryngectomy include massive laryngeal dysfunction associated with aspiration and recurrence after radio(chemo)therapy, resulting in salvage surgery. The surgical procedure including neck dissection is highly standardised and safe. The resulting aphonia can be compensated by functional rehabilitation (e.g., voice prosthesis) associated with a significant quality of life improvement. This article presents an overview of indications, preoperative diagnostics, surgical procedures, including new developments (robotics), possible complications, the choice of adjuvant treatment, alternative therapeutic approaches, rehabilitation and prognosis. In summary, total laryngectomy still represents a relevant surgical procedure in modern head and neck oncology.

## 1. Background

Total laryngectomy is the surgical removal of the larynx. It was first performed on a patient with laryngeal cancer by Christian Albert Theodor Billroth in Vienna on 31 December 1873 [1]. Ever since then, advanced (recurrent) laryngeal and hypopharyngeal cancer have been the main indication for laryngectomy.

Worldwide, nearly 200,000 laryngeal cancer cases are newly diagnosed per year [2]. It is more common in men than women, with a ratio of 7:1. The mean age of onset is 63 in women and 66 in men [3]. Relevant risk factors associated with the disease include the consumption of alcohol and tobacco, but also viral infections, reflux, environmental influences and genetic factors [2]. The consumption of both alcohol and tobacco seems to have a synergistic effect [4]. Laryngeal carcinoma is a recognised occupational disease with asbestos and uranium exposure [5]. Compared with oropharyngeal cancers, the human *papillomavirus* (HPV) status has a subordinate role in laryngeal cancer; the prevalence is about 6% [6]. Its relevance is, however, under discussion as HPV-positive laryngeal cancer seems to have a more favourable prognosis [7], currently without being of relevance for treatment decisions [8]. Hypopharyngeal cancer has the same risk factors but, with an incidence of about 1/100,000, is considerably less common than laryngeal cancer. The lack of symptoms until a later stage and the resultant late diagnosis, together with early lymphatic spread, add up to a poorer prognosis [9,10]. It is well known that cigarette smoke increases the relative risk of developing a supraglottic tumour, while alcohol promotes hypopharyngeal tumours [11,12]. A screening programme for malignant tumours of the upper aero-digestive tract is easy to perform in practice [13], but by consensus is not recommended at the present time as there is no evidence to suggest that it lowers the mortality [8].

## 2. Diagnostic Work-Up

**Figure 1:**
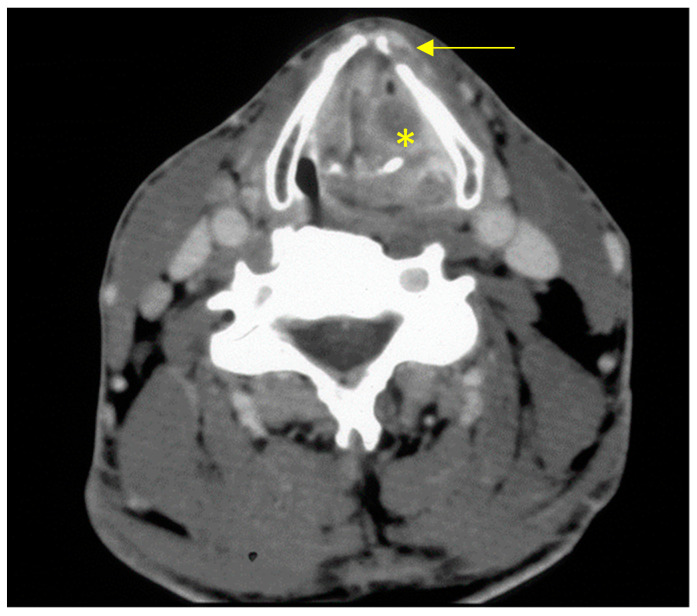
Image from contrast-enhanced CT of the neck from a patient with transglottic cT4a squamous cell carcinoma of the larynx (yellow asterisk = tumour, arrow = cartilage infiltration).

Potential symptoms of a patient with laryngeal cancer are hoarseness, dyspnoea and dysphagia [14]. A hoarse voice lasting more than four weeks should be examined endoscopically [8]. Precancerous conditions such as persistent vocal cord leucoplakia should be completely excised if possible (excision biopsy), as some 20% of patients develop laryngeal cancer within five years [15]. If laryngeal cancer is suspected, the National Comprehensive Cancer Network guidelines recommend besides history taking, a clinical ENT examination, a biopsy of the primary tumour as part of a panendoscopy under anaesthetic, contrast-enhanced computed tomography (CT, Figure 1)/magnetic resonance imaging (MRI) of the neck, CT of the chest, possibly positron emission tomography (PET) with CT (PET-CT) for UICC stages III-IV, a dental examination, audiometry, assessment of speech and swallowing, possibly lung function tests, and presentation to a multidisciplinary tumour board [16]. A national guideline on laryngeal cancer restricts the indication for imaging technique to tumours in the supraglottis, subglottis and anterior commissure, and those with impaired vocal cord mobility [8]. MRI offers better resolution of the soft tissues, which is important for assessing the laryngeal cartilage; however, CT is often preferred because it is more readily available and has fewer motion artefacts. PET-CT is well-suited to the preoperative detection of occult tumour formation, but its use is reserved for patients with advanced tumour stages (T3/4, N2/3) because of its high costs and low availability [16,17]. Even so, PET-CT is being considered, especially in the English-speaking world, as an alternative to elective neck dissection after primary radiochemotherapy (RCT) for advanced laryngeal cancer [18,19,20]. Various working groups have also discussed the use of sentinel node biopsy for cN0 neck as an alternative to elective neck dissection [21,22,23]. Liver metastases are rare (<2%) at the first diagnosis of head or neck cancer [24]; however, risk-adapted abdominal imaging using ultrasound or CT seems worthwhile.

After diagnostic work-up, laryngeal and hypopharyngeal cancers are categorised by the current 8th TNM classification, and staged according to the UICC (Union internationale contre le cancer) criteria [25].

### 2.1. Indication for Total Laryngectomy

Total laryngectomy is indicated for advanced locoregional laryngeal and hypopharyngeal cancers if a partial resection would not remove the primary tumour completely (R0 resection) or would result in significant impairment of function, the patient prefers the operation, and alternative therapeutic approaches such as radio(chemo)therapy alone are inappropriate or are not desired as first-line treatment [26]. To be more precise, there may be extensive tumour involvement of the larynx, penetration of the tumour through the laryngeal skeleton, hypopharyngeal tumours with invasion of the larynx, or extensive tumour recurrence after surgery or prior radio(chemo)therapy. Subglottic cancer presents a special case that may require laryngectomy even in early stages.

Furthermore, a laryngectomy may be indicated for functional reasons, whether an incurable laryngeal fistula or dysphagia and aspiration after transoral or open partial resection of the larynx. In specific cases, laryngectomy may also be required for non-cancer laryngeal conditions (trauma, chemical burns, etc.) [27]. In summary, the main indications for laryngectomy are advanced laryngeal and hypopharyngeal cancers, laryngeal dysfunction (aspiration) and extensive recurrence after previous radio(chemo)therapy.

An important term in this context is “salvage laryngectomy”, originally used for laryngectomy performed for treatment failure with ongoing primary radio(chemo)therapy. Although not strictly accurate, it is frequently used for the surgical procedures necessary for recurrences after radio(chemo)therapy or (combined) cancer surgery [28]. Finally, laryngectomy seems to be more cost effective than organ preservation (Beck, A.J.C.C.; van Harten, W.H.; van den Brekel, M.W.M.; Navran, A.; Retèl, V.P. Cost-Effectiveness of Surgery Versus Organ Preservation in Advanced Laryngeal Cancer. *Laryngoscope* **2021**, *131*, E509–E517.), which might be of relevance in some areas in the world. On the other hand, post-laryngectomy care (accessibility of heat/moisture exchanger (HME)/voice prosthesis, trained speech therapists) may be less or not at all available in some areas.

The indication for laryngectomy and the associated treatment are determined by a tumour conference (including input from self-help groups) as an interdisciplinary multimodal treatment concept. Treatment decisions are made on the basis of established national and international standards [8].

### 2.2. Surgical Technique for Total Laryngectomy

**Figure 2:**
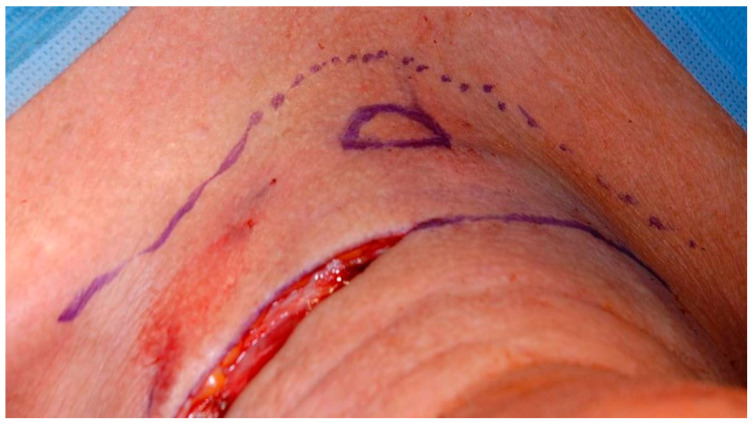
Separation of the tracheostomy cut from the U-shaped incision in total laryngectomy (view from above).

A U-shaped incision is started from the mastoid on both sides, continued down on the anterior border of the sternocleidomastoid muscle and joined over the suprasternal notch. A flap of skin and platysma (apron flap) is dissected out to above the hyoid and folded upwards. As a rule, a radical, modified radical, or selective neck dissection is performed (except functional laryngectomy), depending on the local lymph node involvement [29]. It is followed by dissection of the infra- and suprahyoid muscles and may be associated with the removal of the hyoid bone if necessary. As long as the preoperative diagnostic investigations have not shown any extension towards the thyroid gland, the isthmus is divided and the free ends of the lobes oversewn to channel the access towards the trachea. Should there be thyroid gland invasion or a subglottic tumour, the gland is resected on at least the affected side. The laryngeal vessels are ligated. The fibromuscular tube of the pharynx is separated from the laryngeal skeleton: first, the pharyngeal constrictor muscles are dissected off at various levels. The pharynx is then opened transversely at the level of the hyoid (pharyngotomy). The epiglottis is retracted inferiorly with a clamp. The larynx can now be resected with a macroscopic resection margin of at least 0.5 cm around the tumour [30]. For this purpose, the pharyngeal incision can be extended inferiorly on both sides of the epiglottis to the level of the cricoid. The retro-cricoid mucosa is bluntly dissected off the posterior of the cricoarytenoid muscle. The proximal trachea is bluntly separated from the oesophagus. The exposed larynx can now be brought out through a transverse tracheal incision and by joining the bilateral pharyngeal incisions. Clear resection margins can be verified by frozen section during the operation. For tracheostomy, the tracheal ostium is sutured to the skin in the midline, at the inferior pole of the initial skin incision, and later to the platysma mucocutaneous flap. Alternatively, a second transverse skin incision can be made a few centimetres superiorly or inferiorly to the initial U-shaped skin incision [31], as an attempt to ensure greater stability of the tracheostomy apart from the major skin incisions (Figure 2).

**Figure 3:**
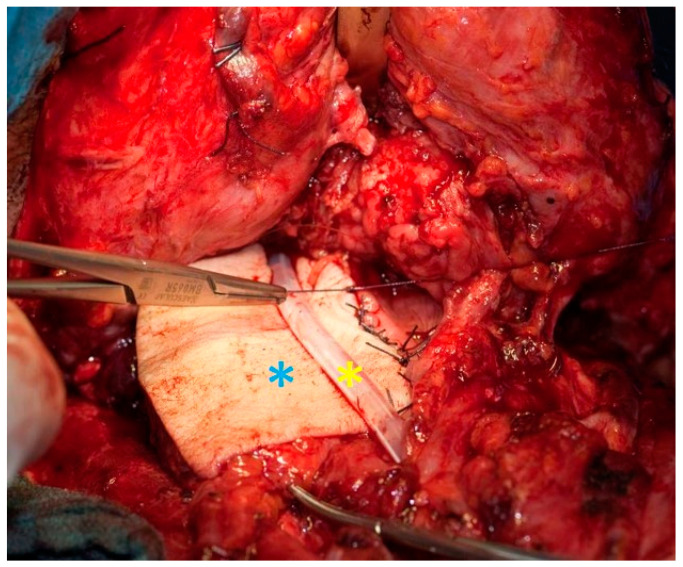
Laryngopharyngectomy with pharyngeal reconstruction by a free microvascular anastomosed radialis graft (blue asterisk). Feeding tube in the middle (yellow asterisk).

The muscles of the superior oesophageal sphincter or the lower part of the inferior pharyngeal constrictor muscle are carefully divided with a scalpel (myotomy), in order to ensure a sufficient postoperative width of the swallowing tract. Variations of the myotomy (unilateral, bilateral or unilateral with neurectomy of the nerves innervating the contralateral side) are possible. Depending on the patient′s wishes and other considerations with respect to speech rehabilitation, a fistula can be made between the trachea and proximal oesophagus, and a speaking valve inserted into it. The valve is inserted with the manufacturer′s specific system, which usually consists of a trocar, an aid to insertion. The speaking valve is selected after measuring the shunt length and width. The pharynx is closed in layers. Particular care is needed to reduce the risk of a fistula. The mucosa is then inverted with a submucous suture. The first suture can be oversewn with a second suture. The prelaryngeal muscles are then sutured over the laryngeal mucosa. The skin is closed in layers, with suction drains placed on both sides. In the case of extensive carcinoma when primary closure of the pharynx is not possible with a single suture—as otherwise the lumen would be functionally too small (≥4 cm mucosa)—a radialis graft or pectoralis major flap can be used to augment the lumen [27,28,32,33] (Figure 3).

### 2.3. Transoral Robotic Laryngectomy

Minimally invasive transoral robotic surgery for laryngectomy (TORS-LE) is not yet established as standard treatment in routine clinical practice. It requires the surgeon to undergo training in the use of robots, as well as planning enough time and justifying the high material costs. In addition, it is important to select the right patients. TORS-LE is performed through a comparatively small tracheostomy skin incision with a transoral approach [34,35]. Robot-assisted laryngectomies have been carried out successfully and with few complications in studies with small case numbers [36,37,38]. TORS-LE appears suitable for patients with advanced laryngeal cancer if there is no invasion of the prelaryngeal soft tissues or retrocricoid region [35]. Should neck dissection be required, it can likewise be performed endoscopically or carried out 2–3 weeks later [35]. The idea is that the smaller access pathway of TORS-LE will reduce the risk of complications such as a salivary fistula [34]. At the present time, there are too few reliable data on salvage TORS-LE, and it has been shown that postoperative complications such as fistulas still have to be considered even with this procedure [38]. In addition, there are some reservations about whether the impeccable hygienic preparation of such small instruments is possible [34]. Furthermore, in a few cases of TORS-LE, the procedure has had to be switched to open surgery to allow the complete resection of the tumour when it could not be adequately exposed [38]. Further studies are required for a satisfactory evaluation.

### 2.4. Tracheostomy before Total Laryngectomy

At the present time, it is recommended that a tracheostomy before laryngectomy should be avoided if at all possible and, for example, tumour-induced dyspnoea treated by debulking the tumour mass during an initial panendoscopy [8], since preoperative tracheostomy seems to increase the risk of tracheostomy recurrence [39]. However, this issue is a subject of controversy [40]. Furthermore, tracheostomy-specific complications that may need an extension of therapeutic measures have been reported. Besides the general surgical risks, these complications include pneumothorax, emphysema, cardiac arrhythmias, aspiration and pneumonia, development of a fistula, tracheal stenosis and tracheomalacia [41]. On the other hand, before or during primary radio(chemo)therapy, a tracheostomy may become inevitable due to swelling.

### 2.5. Percutaneous Endoscopic Gastrostomy

A percutaneous endoscopic gastrostomy (PEG) has a subordinate role before and after laryngectomy and more often comes into consideration when there is persistent dysphagia and aspiration after partial laryngectomy or primary radio(chemo)therapy [42]. In some circumstances, however, prophylactic gastrostomy tubes may be useful in selected cases with an expected increased tendency to fistula formation (e.g., salvage laryngectomy) and dysphagia, in the same way as the prophylactic gastrostomy that is sometimes already practiced prior to radiotherapy of locally advanced laryngeal and hypopharyngeal cancers [43].

### 2.6. Neck Dissection

Laryngeal carcinomas drain particularly into level II–IV [28], hypopharyngeal cancer into level II–V, although tumour invasion of the apex of the piriform sinus in particular may also involve lymph nodes at level VI [44]. Depending on the site of the tumour, the lymph node status in the neck, and the therapeutic measures already taken, there are various types of neck dissection based on the goal and proposed extent. Neck dissection is considered “elective” if it is indicated in the case of cervical lymph nodes that are clinically unremarkable (cN0). With positive cervical lymph nodes (cN1-3), neck dissection may be “therapeutic”. If radiotherapy or neck dissection has already been carried out or was unsuccessful and there is still an indication or a new indication (r/yxN1-3) for neck dissection, this is designated a “salvage” neck dissection. Based on the level of the resected nodes and the structures contained therein, neck dissection can be subdivided into superselective, selective, modified radical, radical and supraradical [29]. If neck dissection is carried out before the planned definitive treatment, it is referred to as an “up-front” neck dissection [29]; however, this procedure is not an issue in routine clinical practice. Neck dissection may be unilateral or bilateral, depending on the site and extent of the tumour.

Patients with unilateral subglottic or advanced glottic squamous cell carcinoma and cN0 status should have at least a unilateral elective neck dissection as part of the primary laryngectomy, while patients with supraglottic, hypopharyngeal or approximately midline tumours should have a bilateral elective neck dissection, in order to minimise the risk of recurrence from clinically occult metastases, especially if there is no plan for adjuvant radio(chemo)therapy to cover the lymphatic drainage territory [28,29,45]. On the other hand, there is no general recommendation for elective neck dissection in the case of cT1/cT2 glottic cancer with cN0 status [45]. Dissection of levels II-IV and VI is recommended for advanced laryngeal and hypopharyngeal tumours that extend below the glottis, otherwise levels II-IV usually suffice [46]. With cN1-3 status, modified radical neck dissection should be carried out together with a planned laryngectomy [45]. According to the American Society of Clinical Oncology guidelines, neck dissection may be considered in patients beyond cN2 status who are receiving radio(chemo)therapy, irrespective of their response to treatment [45]. A salvage neck dissection after primary RCT should not be carried out unless lymph node metastases are still demonstrated on PET-CT; otherwise, the advice is for clinical monitoring [20]. PET-CT has become an important tool for evaluating whether neck dissection is necessary. With a cN0 neck, patients can forgo elective neck dissection after primary RCT if no metastases are seen on PET-CT scans [20]. Additionally, in cases with ≥ N2 neck status, PET-CT scan-guided watchful waiting is being discussed as an alternative to salvage neck dissection after RCT [8,20]. Patients with suspicious-looking lymph nodes on PET-CT scans still have to undergo neck dissection [19].

## 3. Adjuvants

Beyond UICC stage III, adjuvant radio(chemo)therapy should be given after primary surgical procedures for laryngeal and hypopharyngeal cancer [16,47], especially if there are risk factors for early recurrence with extranodal growth, blood or lymph vessel invasion, perineural growth and narrow resection margins [28]. Every effort should be made that patients with histopathological extranodal growth or a narrow resection margin (′close margin′ or <5 mm) do not receive radiotherapy alone but rather radiotherapy and platinum-based chemotherapy [48]. Adjuvant therapy should be started within six weeks after surgery [16] and completed 14 weeks after the operation; any further delay has a negative effect on survival [49]. The current version of the laryngeal cancer guideline gives consensus-based exceptions with respect to the adjuvant therapy of stage III laryngeal cancer: no adjuvant treatment should be given to pT3 pN0 cM0 glottic cancer without the assumption of an increased risk of recurrence (resection margin ≥5 mm, ≥10 tumour-free lymph nodes on the operated side of the neck); furthermore, dispensing with adjuvant therapy may be considered in the absence of risk factors with supraglottic pT3 pN0 cM0 cancer [16]. The adjuvant radiation dose at the site of the tumour can be adapted to between 56 and 66 Gy, depending on the risk, and even higher (70 Gy) with R1 status [16]. A dose of between 46 and 56 Gy is selected for the lymph drainage territory [49]. Depending on the fractionation schedule, treatment generally lasts 6–7 weeks. Cisplatin may be administered as part of concomitant RCT; for example, 100 mg/m^2^ every three weeks for a total of 2–3 cycles [16,50]. Principally, adjuvant treatment should be discussed in tumour board meetings and should be tailored to patients (not only to TNM status).

### 3.1. Radio(chemo)therapy as an Alternative

Studies by Greg Wolf in Ann Arbor, Michigan, were one of the reasons for developing a non-surgical form of treatment with the combination of chemotherapy and radiotherapy [51]. At the present time, should the decision for primary radiotherapy be made in line with the patient’s wishes to preserve the larynx, because of contraindications to surgery, or with the likelihood of an incomplete resection (e.g., with T4b laryngeal/hypopharyngeal cancer [8]), treatment should be combined with concomitant chemotherapy—and also immunotherapy in exceptional cases—in order to increase patient survival [16,47,52,53]. Patients over the age of 70 may be given radiotherapy alone if there are justified concerns about the tolerability of combined treatment [8].

As opposed to radiotherapy alone or induction chemotherapy followed by radiochemotherapy (RCT), concomitant RCT offers the advantage of laryngeal preservation [47,52]. It should be noted that non-cancer-associated study-specific deaths were highest in the concomitant RCT group, which may have been responsible for the overall survival not being significantly better than in the other two alternative groups mentioned above [47].

In the absence of contraindications, cisplatin-based chemotherapy, e.g., with 5-fluoruracil, is to be recommended. Alternatives include the use of carboplatin with/without 5-fluoruracil, of mitomycin C with/without 5-fluoruracil, or of cetuximab simultaneously with the radiotherapy [47,54,55]. Retrospectively, however, cetuximab has not been shown to be a comparable alternative to cisplatin with respect to overall and progression-free survival (even taking the propensity score into account [56]) [56,57,58]. So far, immune checkpoint inhibitors are approved for recurrent and metastatic or palliative treatment and investigated in clinical studies for curative settings [59,60], (integrate Burtness B.; Harrington, K.J.; Greil, R.; Soulières, D.; Tahara, M.; de Castro, G.; Psyrri, A.; Basté, N.; Neupane, P.; Bratland, Å.; at el. Pembrolizumab alone or with chemotherapy versus cetuximab with chemotherapy for recurrent or metastatic squamous cell carcinoma of the head and neck (KEYNOTE-048): a randomised, open-label, phase 3 study. *Lancet* **2019**, *394*, 1915–1928). The intensity-modulated radiation dose for primary treatment is about 70 Gy at the tumour site and between 50–60 Gy in the electively irradiated lymph drainage territory [47,61]. The duration of RCT lasts, for example, seven weeks with non-accelerated fractionation of 70 Gy to 2 Gy per individual dose and two to three cycles of cisplatin (100 mg/m^2^ three-weekly) [16,47].

With respect to prognosis, primary RCT is not superior to laryngectomy with subsequent adjuvant therapy [45,51], whereby complications are seen with both approaches. Late, extremely toxic effects of concomitant RCT occur particularly in elderly patients and patients with high T status/UICC stage III/IV laryngeal and hypopharyngeal cancers (>40%) [62]. Such complications include Radiation Therapy Oncology Group/European Organisation for Research and Treatment of Cancer (RTOG/EORTC) grade III and IV radiotoxicity, dysphagia persisting for more than 180 days, and the necessity to bypass oropharyngeal swallowing structures with a PEG tube for at least two years [63]. In the RTOG 91–11 study, the cumulative 10-year grade III–V toxicity rate with concomitant RCT was 33% [47]. Specifically, this means atrophy, fibrosis, induration, ulceration and necrosis of the skin, mucosa or parenchyma, xerostomia, dysphagia, neuropathies, pain, impaired vision, pneumonitis, cardiomyopathy and injury to the liver and kidneys, as well as to the bone and cartilage. One in five patients with advanced (UICC stage III/IV) glottic or supraglottic laryngeal carcinoma needs a laryngectomy for residual or recurrent tumour within ten years of primary concomitant RCT [47].

A further approach to advanced squamous cell carcinoma of the head and neck is induction chemotherapy prior to concomitant RCT [64]. The aim is to improve the baseline situation by giving induction chemotherapy before the definitive RCT. Further reasoning is to identify patients who will probably respond well to RCT (chemoselection) and to treat the others (non-responders) primarily by surgery, in order to prevent increased morbidity after an otherwise impending salvage laryngectomy in the latter group [65]. As a rule, studies on induction chemotherapy have used the TPF regimen (docetaxel (T), cisplatin (P), 5-fluoruracil (F)) [64,66]. The phase II DeLOS trial, which enrolled patients with advanced stage III/IV laryngeal or hypopharyngeal cancer that could still effectively be cured by laryngectomy, compared the TPF regimen and subsequent radiotherapy with the TPE regimen (5-fluoruracil replaced by cetuximab) and subsequent radiotherapy. With respect to the two-year survival, no benefit was found in comparison with the study arm without cetuximab [67]. Overall, the study showed no advantage of additional induction chemotherapy over concomitant radiotherapy alone in terms of recurrence-free or overall survival. It should also be noted that induction chemotherapy with subsequent RCT is more often terminated prematurely due to adverse reactions, so that the initially intended doses of chemotherapy and radiotherapy often cannot be achieved [66]. For this reason, neoadjuvant chemotherapy should at best be used for selection purposes, or in the context of clinical studies [8].

### 3.2. Surgery or Radiochemotherapy?

The choice of an appropriate therapeutic regimen is not a trivial matter and should be made within the context of an interdisciplinary case conference [8]. Patients with T2/T3 laryngeal or hypopharyngeal cancer in whom partial laryngectomy is unlikely to achieve complete tumour resection with functionally acceptable results but who still have (partial) function of the larynx prior to treatment are particularly well-suited to primary RCT [68]. Even so, retrospective data show that patients with T3 laryngeal or hypopharyngeal cancer who undergo primary laryngectomy, at the expense of larynx preservation, followed by adjuvant radio(chemo)therapy benefit with respect to overall survival compared with those undergoing primary RCT (five-year survival: 65% vs. 44%) [69]. In a selected patient population, primary RCT may have comparable success [70]. Nevertheless, there are still no data from prospective randomised trials that confirm the superior survival of patients undergoing primary surgical treatment in the above-mentioned scenario, as was found retrospectively. The bias induced by a retrospective approach has to be taken into consideration, as, for example, patients with reduced performance status tend to be allocated into a conservative treatment arm. Accordingly, in order to ensure an acceptable outcome when larynx-preserving treatment is contemplated for cT3 carcinoma, apart from considering the patient’s wishes, it is particularly important to rule out stage T4 by radiology, to assess whether the proposed radiotherapy clinic and the patient are suited to carrying out the required treatment in full, and to determine whether the patient can be trusted to attend for regular after-care [71]. Otherwise, laryngectomy should be preferred even though subsequent adjuvant therapy may also be necessary in this case [71]. With T4a cancers, primary surgical treatment is clearly preferred for laryngeal and hypopharyngeal cancer [10,68,69,71,72]. The reasons for this relate to prognosis and function. The large tumour volume corresponding to this stage and the possible necrotic or hypoxic components of the tumour mass are responsible for an increased resistance to radiation and thus a poorer response to treatment [73,74]. In addition, T4a tumours carry the risk of laryngeal perichondritis with an increased need for secondary laryngectomy. Furthermore, advanced laryngeal and hypopharyngeal cancers often cause relevant functional problems such as dyspnoea, dysphagia and aspiration [14]. Once again, these can be treated by laryngectomy. However, primary RCT remains for T4b tumours which are not operable. In addition, it is unlikely that radio(chemo)therapy can downstage/downsize a tumour in that way that total laryngectomy remains possible afterwards [75]. Irrespective of these considerations, the feasibility of each type of treatment has to be checked, taking into account the age, comorbidities and the wishes of the patient in the individual case. Ultimately, primary laryngectomy is worthwhile particularly in patients with T4a laryngeal or hypopharyngeal cancer and marked pre-treatment laryngeal dysfunction such as an inability to swallow.

### 3.3. Salvage Laryngectomy

If, after primary radio(chemo)therapy, there is residual tumour or a recurrence in the absence of distant metastases, salvage laryngectomy can be considered together with elective or salvage neck dissection, depending on the lymph node status [28]. With positive lymph nodes, neck dissection is basically indicated [29]; elective neck dissection is also usually carried out with T3/T4 and supraglottic tumours [76]. These cases carry a high risk of occult metastases (T1: 9%; T2: 10%; T3: 16%; T4 34%; supraglottic T1-4 28%, supraglottic T4: 50%) [77]. Even the risk of occult metastases in clinically unsuspicious cervical lymph nodes seems to be much lower after primary R(C)T, selective neck dissection (levels IIa, III, IV) is usually included in the salvage procedure [78], since PET-CT seems to have a poor sensitivity in this situation [79]. In general, salvage laryngectomy is usually performed within two years of the primary RCT [47].

**Figure 4:**
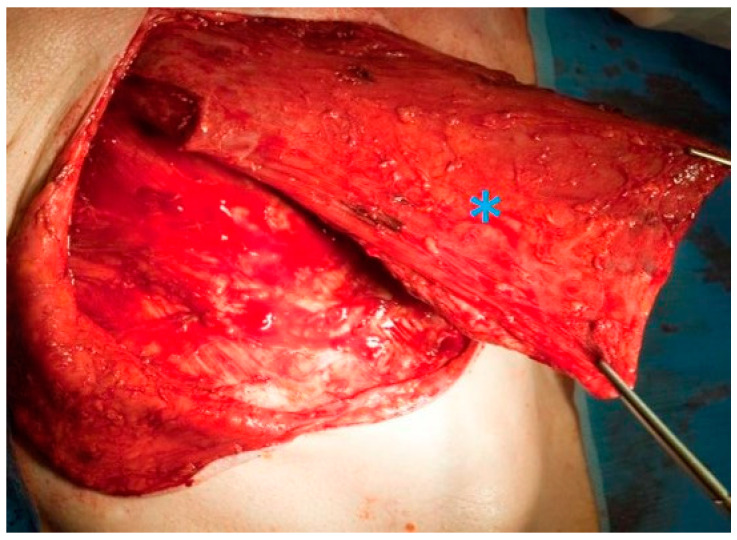
Raising a pectoralis major pedicle flap (blue asterisk) to prevent wound healing problems such as fistula formation in a salvage laryngectomy.

Following concomitant RCT in patients with stage III–IV glottic/supraglottic laryngeal cancer the RTOG 91-11 study (*n* = 182) found that salvage laryngectomy was required in about 20% within ten years [47,53]. Similarly, a study from the Netherlands on T3 laryngeal cancer found that salvage was required in more than 20% of cases within a three-year interval (*n* = 48) [80]. From the surgical point of view, salvage laryngectomy harbours various special challenges. Radiation fibrosis, scarring, and oedematous tissue changes can make the dissection more difficult [81]. As radiotherapy impairs wound healing, it has been shown that the inclusion of well-vascularised tissue from the non-irradiated surroundings (e.g., pectoralis major flap, Figure 4) is beneficial [76].

## 4. Complications of Total Laryngectomy

The complications of laryngectomy can be divided into general surgical risks, specific complications and negative psychosocial consequences. They are closely related to the treatment previously carried out (primary surgical procedure versus salvage operation). Perioperative and postoperative complications occur to a relevant extent with laryngectomy (20–30% [82,83]) and frequently with salvage laryngectomy (up to 60–70% [81]), but are usually well manageable in specialised centres.

General surgical risks include wound infections and delayed wound healing, haemorrhage and haematomas [28]. Wound infections occur in 9%, wound dehiscence in 4% of patients [84]. Considering salvage laryngectomised patients separately, the rate of wound infections is 14% and 9% for wound dehiscence [85]. Postoperative haemorrhage and haematomas occur in less than 10% of patients undergoing laryngectomy (including salvage laryngectomy) [82,85]. The need for an intraoperative or postoperative blood transfusion within the first three days is about 15% [84]. Pneumonia and sepsis have each been described in 5% [84]. Death occurring postoperatively has been reported in about 1% of patients [82,86]. The underlying reasons are pneumonia [87], myocardial infarction [53], or rupture of the common or external carotid artery [87]. The rare complication of carotid artery rupture is probably due to the subacute exposure of the vessel with a disorder of wound healing such as dehiscence, dislocation of the graft, fistula formation, tissue necrosis or previous radiotherapy [88].

Specific surgical risks are dysphagia and pharyngocutaneous fistulas [28]. The latter occur in about 14% of laryngectomised patients [82,87], with a 2-fold increase after RCT [76,89,90]. The use of a pectoralis major pedicle flap or radialis graft has a protective effect [28,76,85].

**Figure 5:**
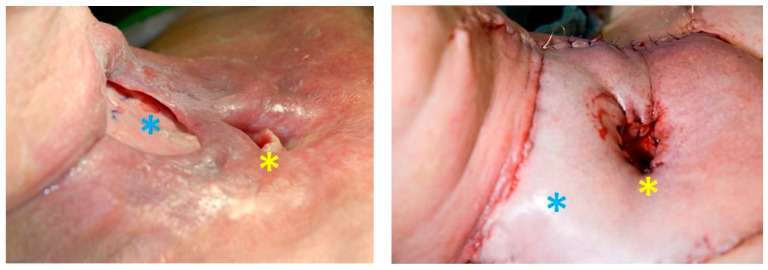
Patient with a pharyngocutaneous fistula (left, blue asterisk = fistula, yellow = stoma) after salvage laryngectomy and closure of the fistula with a deltopectoral pedicle flap (right, blue asterisk = flap, yellow = stoma).

The American Society of Health-System Pharmacists guideline recommends prophylactic ampicillin/sulbactam or cefazolin/cefuroxime and metronidazole to reduce wound infections [91]. This should be started preoperatively (30 min before incision [91]) and continued for 48 h if a vascularly anastomosed graft is required, otherwise for only 24 h [92]. Spontaneous fistula closure is seen after an average of approximately 25 days in about 90% of patients. Looking solely at salvage laryngectomised patients, closure takes a mean of 50 days [89]. If fistula closure does not occur spontaneously, surgery (e.g., tissue transfer as shown in Figure 5) and multiple-layer wound closure may be required [81], possibly with the temporary use of a salivary bypass tube [82,90]. Particularly after salvage laryngectomy, dysphagia has also been recorded in 3–30%, pharyngeal stenosis in 14% (usually after flaps or grafts) and breathing difficulties in 4–9% of cases [85]. Postoperative pharyngo-oesophageal strictures can usually be treated by bougies or alternatively with botulinum toxin [28]. Surgical revision is required in about 12% of cases [84]. Possible complications after fashioning a tracheo-oesophageal fistula for speech rehabilitation include periprosthetic or transprosthetic leakage, dislocation and excessive microbial colonisation of the prosthesis [28]. Furthermore, depending on the extent of a necessary neck dissection, other adverse effects such as accessory nerve alteration may result [85]. In addition, bothersome but not life-threatening symptoms are frequently reported: xerostomia, dysgeusia, mucositis, burning sensation of the skin, chronic pain, malaise, fatigue, trismus, neck stiffness, lymphoedema, dysaesthesia, mucus secretion and tracheal encrustation, which are also well-known as typical effects of radiation. Psychological and social complications such as depression, social isolation and financial problems due to the consecutive reduction of fitness to work and unemployment are of relevance to the patients.

Predictors of possible complications with laryngectomy are described in the following and are important to note. Risk factors for the development of a pharyngocutaneous fistula include chronic obstructive pulmonary disease, preoperative haemoglobin <12.5 g/dL, the necessity of blood transfusion, high T status, R1/2 resection and the performance of neck dissection [93]. In particular, previous radiotherapy (<12 months before [94]) has a negative effect on the fistula rate; additional chemotherapy does not seem to have any negative effects on this rate [85]. Hypothyroidism is also associated with an increased rate of fistulas [95]. Sarcopenic patients have more disorders of wound healing and require early nutritional optimisation [96]. Furthermore, patients with diabetes have a higher rate of wound infections but no higher mortality [97]. Lastly, complications arise more frequently with hypopharyngeal cancer than with laryngeal cancer: fistulas and wound infections were seen twice as often with hypopharyngeal cancer [90]. However, morbidity and mortality are significantly reduced in centres with higher case numbers [98].

## 5. Prognosis after Laryngectomy

Survival after laryngectomy depends on the TNM stage and the site of the laryngeal/hypopharyngeal primary tumour, as well as comorbid conditions. Patient individualized prognosis can be calculated, taking comorbidity into account (see https://www.oncologiq.nl; accessed on 19 March 2021). Head and neck surgeons and oncologists can benefit from this information when discussing prognosis with their patients. Mean overall five-year survival is 60% with laryngeal cancer and 50% with hypopharyngeal cancer; it is 55% with T4 laryngeal cancer and 35% with T4 hypopharyngeal cancer. The lowest survival rate after total laryngectomy is seen with subglottic tumours (40%) and retrocricoid tumours (15%); the highest survival rate is found with glottic cancer (70%), also due to its early symptoms [90]. Survival is reduced by half in patients with hypopharyngeal squamous cell carcinoma and involvement of level VI, compared with patients in whom level VI is not involved [44]. Recurrence after laryngectomy in terms of locoregional recurrences, lymph node and distant metastases, or a second cancer occurs in about 30% of patients after one year on average [28]. Therefore, imaging during oncology after-care is particularly important in these cases. Should salvage laryngectomy be required after primary RCT, the survival in these patients is lower than in those who are recurrence-free [53]. With stage III/IV laryngeal cancer, the five-year survival falls to 37% [76]. In addition, patients should be encouraged to stop their consumption of noxious substances, as this can further impair the prognosis [45].

## 6. Rehabilitation

Following laryngectomy, the patients’ goal is to learn how to participate self-sufficiently in everyday life and at work in the long term. After a laryngectomy, a reduction in earning capacity of 100% is assumed for up to five years and afterwards of at least 70% with good phonation [99].

For rehabilitation, patients have to practice their own tracheostomy/tube care, swallowing, breathing and communicating. These exercises should be started in hospital [8] and then consolidated with domiciliary nursing care and speech and language therapists. Laryngectomised patients need special equipment for home care, and this is usually requisitioned during the hospital stay. It includes the appropriate tracheal tube, a heat/moisture exchanger (HME), possibly with a speaking valve, inhaler, suction device, and various cleaning and care products [1]. After leaving hospital, the patient usually undergoes intensive training in a specialised rehabilitation facility if feasible. Supportive measures can be adapted to need: physiotherapy, psychological support, nutritional, social and possibly addiction counselling, as well as participation in self-help groups. Here they should be encouraged to focus on the positive aspects and actively seek social support [100]. This may reduce their anxieties and allow them to make a more reasoned decision about possible treatment options [101].

**Figure 6:**
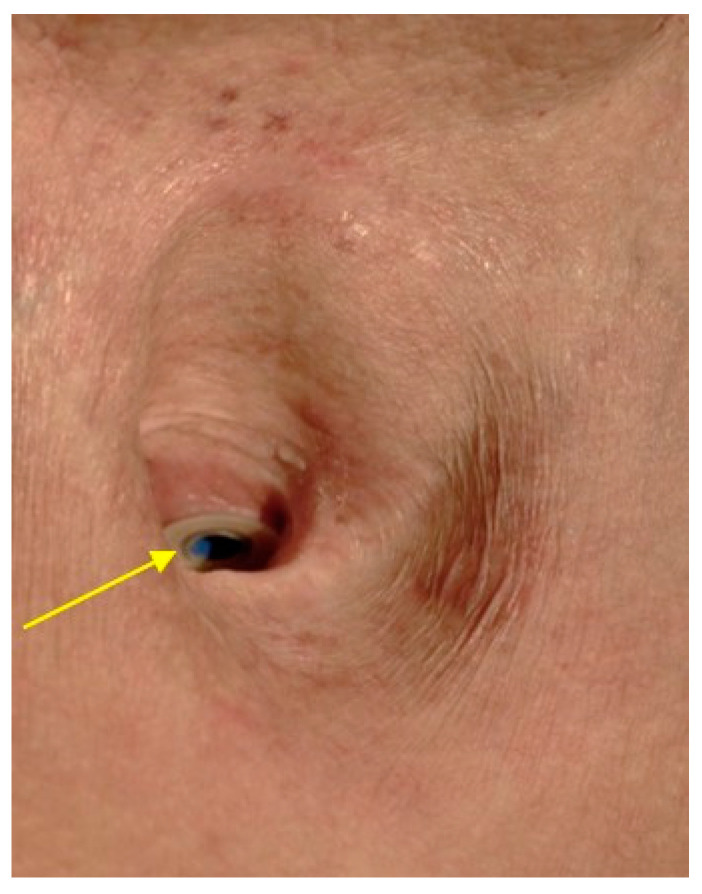
Tracheostoma with voice prosthesis (yellow arrow), inserted in the posterior tracheal wall and allowing for voice rehabilitation.

The preferred variant of speech rehabilitation is phonation through a primary or secondary tracheo-oesophageal fistula with a voice prosthesis or shunt valve to use the oesophagus and pharynx as resonators (Figure 6) [28]. This valve can be combined with a tracheostomy valve for a “finger-free” and thus less conspicuous speech process [1]. In Western Europe, the majority of laryngectomised patients receive a voice prosthesis [102]. The primary surgical fashioning of a tracheo-oesophageal fistula requires comparatively little time, has few complications, and promotes rapid speech rehabilitation within a few days of the laryngectomy, giving easily understandable phonation in 90% of cases [102], even with irregularly shaped tracheostomies (e.g., pronounced sternal attachment which should be considered during primary surgery) if a suitable epithesis is used [103].

Voice prostheses with a diameter of 16–22.5 Fr and a length of 4–12.5 mm are available from various manufacturers. In addition to those extra-large flanges, coatings, e.g., with silver oxide to prevent biofilm formation, are offered. There exist also prostheses with magnets as an option for patients with repetitive rapid leakage. Further options for speech rehabilitation are training in oesophageal speech or the use of an electrolarynx. Surgical procedures to build a pseudoglottis are described, but have not entered the status of a clinical routine [104].

Olfaction can be re-learned with the “polite yawning” technique [105]. Yawning is carried out with closed lips creating a negative pressure in the mouth and throat, which can induce airflow through the nose. In addition, there is a theoretical possibility of a laryngeal bypass system with tubes joining the tracheostomy and mouth outside the body, once more allowing breathing through the nose and even making it possible to go diving wearing specially designed masks [106]. The use of an HME as an artificial nose is recommended to protect the lungs and tracheal mucosa [1,105].

## 7. Quality of Life after Total Laryngectomy

The general state of health of laryngectomised patients can mostly be described as only “satisfactory” on a six-point scale. In particular, there are restrictions in social life. Physical and mental stress come from a reduced sense of smell, stigmatising or altered voice, insecurity in one’s personal bearing, dry mouth, cough, fatigue, shortness of breath, sleeplessness, financial difficulties and fears for the future [100,107,108]. The actual psychosocial status after laryngectomy (cognition, finances, social contact, eating in company, etc.) is not identical to the hoped-for status but often stagnates below expectations. A corresponding preoperative explanation of the possible consequences is therefore extremely important. Learning appropriate useful coping strategies is important for dealing with the new situation and affects the quality of life [100]. Speech rehabilitation should be carried out with a voice prosthesis whenever possible [109] and is crucial for the patient’s quality of life [14]. It is problematic that the reduced quality of life due to functional damage and mental stress does not seem to improve with time, even after 10 years [107]. Even though there seems to be no significant difference in the overall quality of life [110,111], laryngectomised patients can chew and swallow better [112], suffer less from xerostomia [110,111] or pain [112], those patients undergoing primary RCT can speak better [8,110,111,112] and tend to have fewer difficulties with their social surroundings [110].

In summary, total laryngectomy is a well-established and oncologically reasonable operation carried out as part of multimodal treatment in patients with locally advanced laryngeal and hypopharyngeal cancer. Potential complications, e.g., after salvage surgery can be well-managed in experienced tumour centres. Rehabilitation efforts are made with respect to voice and swallowing in an interprofessional manner.

## References

[1] Kramp B., Dommerich S. (2009). Kanülen und Stimmprothesen. Laryngo-Rhino-Otologie.

[2] https://gco.iarc.fr/today/online-analysis-table.

[3] Wiegand S. (2016). Evidenz und Evidenzlücken zur Chirurgie des Larynxkarzinoms. Laryngo-Rhino-Otologie.

[4] Talamini R., Bosetti C., La Vecchia C., Maso L.D., Levi F., Bidoli E., Negri E., Pasche C., Vaccarella S., Barzan L. (2002). Combined effect of tobacco and alcohol on laryngeal cancer risk: A case–control study. Cancer Causes Control..

[5] Michel O. (2016). Berufskrankheit: Kehlkopfkarzinom. HNO.

[6] Castellsagué X., Alemany L., Quer M., Halec G., Quirós B., Tous S., Clavero O., Alòs L., Biegner T., Szafarowski T. (2016). HPV Involvement in Head and Neck Cancers: Comprehensive Assessment of Biomarkers in 3680 Patients. J. Natl. Cancer Inst..

[7] Davidson S.M., Ko H.C., Harari P.M., Wieland A.M., Chen S., Baschnagel A.M., Kimple R.J., Witek M.E. (2018). Impact of HPV Status on the Prognostic Potential of the AJCC Staging System for Larynx Cancer. Otolaryngol. Neck Surg..

[8] Bootz Singer H. (2019). Leitlinienprogramm Onkologie (Deutsche Krebsgesellschaft, Deutsche Krebshilfe, AWMF): Diagnostik, Therapie und Nachsorge des Laryngeal Carzinomas, Langversion 1.0, 2019, AWMF-Registernummer: 017/076OL. http://www.leitlinienprogramm-onkologie.de/leitlinien/larynxkarzinom/.

[9] Güneş S., Başaran B., Aslan İ. (2018). Surgical treatment of hypopharyngeal cancer. Turk. J. Ear Nose Throat.

[10] Petersen J.F., Timmermans A.J., Van Dijk B.A.C., Overbeek L.I.H., Smit L.A., Hilgers F.J.M., Stuiver M.M., Brekel M.W.M.V.D. (2018). Trends in treatment, incidence and survival of hypopharynx cancer: A 20-year population-based study in the Netherlands. Eur. Arch. Oto-Rhino-Laryngology.

[11] Huang C.-C., Hsiao J.-R., Lee W.-T., Lee Y.-C., Ou C.-Y., Chang C.-C., Lu Y.-C., Huang J.-S., Wong T.-Y., Chen K.-C. (2017). Investigating the Association between Alcohol and Risk of Head and Neck Cancer in Taiwan. Sci. Rep..

[12] Menvielle G., Luce D., Goldberg P., Bugel I., Leclerc A. (2004). Smoking, alcohol drinking and cancer risk for various sites of the larynx and hypopharynx. A case–control study in France. Eur. J. Cancer Prev..

[13] Löhler J., Gerstner A.O.H., Bootz F., Walther L.E. (2013). Incidence and localization of abnormal mucosa findings in patients consulting ENT outpatient clinics and data analysis of a cancer registry. Eur. Arch. Oto-Rhino-Laryngology.

[14] Steuer C.E., El-Deiry M., Parks J.R., Higgins K.A., Saba N.F. (2017). An update on larynx cancer. CA Cancer J. Clin..

[15] Kostev K., Jacob L.E., Kalder M., Sesterhenn A.M., Seidel D.U. (2018). Association of laryngeal cancer with vocal cord leukoplakia and associated risk factors in 1,184 patients diagnosed in otorhinolaryngology practices in Germany. Mol. Clin. Oncol..

[16] Network NCC (2018). NCCN Clinical Practice Guidelines in Oncology: Head and Neck Cancers.

[17] Haerle S.K., Schmid D., Ahmad N., Hany T., Stoeckli S. (2011). The value of 18F-FDG PET/CT for the detection of distant metastases in high-risk patients with head and neck squamous cell carcinoma. Oral Oncol..

[18] Grosse J., Hellwig D. (2018). Update: PET/CT und PET/MRT bei Tumoren des Kopf-Hals-Bereiches. Der Nukl..

[19] Mehanna H., McConkey C.C., Rahman J.K., Wong W.-L., Smith A.F., Nutting C., Hartley A.G., Hall P., Hulme C., Patel D.K. (2017). PET-NECK: A multicentre randomised Phase III non-inferiority trial comparing a positron emission tomography–computerised tomography-guided watch-and-wait policy with planned neck dissection in the management of locally advanced (N2/N3) nodal metastases in patients with squamous cell head and neck cancer. Heal. Technol. Assess..

[20] Mehanna H., Wong W.-L., McConkey C.C., Rahman J.K., Robinson M., Hartley A.G.J., Nutting C., Powell N., Al-Booz H., Robinson M. (2016). PET-CT surveillance versus neck dissection in advanced head and neck cancer. N. Engl. J. Med..

[21] Thompson C.F., John M.A.S., Lawson G., Grogan T., Elashoff D., Mendelsohn A.H. (2012). Diagnostic value of sentinel lymph node biopsy in head and neck cancer: A meta-analysis. Eur. Arch. Oto-Rhino-Laryngol..

[22] Tomifuji M., Shiotani A., Fujii H., Araki K., Saito K., Inagaki K., Mukai M., Kitagawa Y., Ogawa K. (2008). Sentinel Node Concept in Clinically N0 Laryngeal and Hypopharyngeal Cancer. Ann. Surg. Oncol..

[23] Werner J.A., Dünne A.-A., Ramaswamy A., Folz B.J., Lippert B.M., Moll R., Behr T. (2002). Sentinel node detection in N0 cancer of the pharynx and larynx. Br. J. Cancer.

[24] Chen P.G., Schoeff S.S., Watts C.A., Reibel J.F., Levine P.A., Shonka D.C., Jameson M.J. (2014). Utility of abdominal imaging to assess for liver metastasis in patients with head and neck cancer and abnormal liver function tests. Am. J. Otolaryngol..

[25] Poon C., Stenson K. Overview of The diagnosis and Staging of Head and Neck Cancer. https://www.uptodate.com/contents/overview-of-the-diagnosis-and-staging-of-head-and-neck-cancer.

[26] Kaanders J.H.A.M., Lenarz T., Pop L.A.M., Schmoll H.J., de Mulder P.H.M., Marres H.A.M. (2006). Larynxkarzinom. Kompendium Internistische Onkologie.

[27] Werner J.A., Rettinger G., Hosemann W.G., Hüttenbrink K.-B., Werner J.A. (2017). Totale Laryngektomie. HNO-Operationslehre.

[28] Agrawal N., Goldenberg D. (2008). Primary and Salvage Total Laryngectomy. Otolaryngol. Clin. N. Am..

[29] Vahl J., Hoffmann T. (2019). Neck dissection-Surgical treatment of cervical lymphatic drainage pathways. HNO.

[30] Alicandri-Ciufelli M., Bonali M., Piccinini A., Marra L., Ghidini A., Cunsolo E.M., Maiorana A., Presutti L., Conte P.F. (2013). Surgical margins in head and neck squamous cell carcinoma: What is ‘close’?. Eur. Arch. Oto-Rhino-Laryngol..

[31] Schlag P.M., Hartmann J.T., Budach V. (2011). Weichgewebetumoren.

[32] Bonkowsky V., Strutz J., Strutz J., Mann W.J. (2017). Laryngektomie. Praxis der HNO-Heilkunde, Kopf- und Halschirurgie.

[33] Fagan J. (2016). Total Laryngectomy. Open Access Atlas Otolaryngol. Head Neck Oper. Surg..

[34] Schuler P.J., Boehm F., Schild L.R., Greve J., Hoffmann T.K. (2021). Robotik in der Kopf-Hals-Chirurgie. HNO.

[35] Schuler P.J., Hoffmann T.K., Veit J.A., Rotter N., Friedrich D.T., Greve J., Scheithauer M.O. (2016). Hybrid procedure for total laryngectomy with a flexible robot-assisted surgical system. Int. J. Med. Robot. Comput. Assist. Surg..

[36] Krishnan G., Krishnan S. (2017). Transoral Robotic Surgery Total Laryngectomy: Evaluation of Functional and Survival Outcomes in a Retrospective Case Series at a Single Institution. ORL.

[37] Lawson G., Mendelsohn A.H., Van Der Vorst S., Bachy V., Remacle M. (2012). Transoral robotic surgery total laryngectomy. Laryngoscope.

[38] Smith R.V., Schiff B.A., Sarta C., Hans S., Brasnu D. (2013). Transoral robotic total laryngectomy. Laryngoscope.

[39] Sheng X., Zhang S., Song X., Chen L., Luo X. (2013). Meta-analysis on the risk factors for stomal recurrence after total laryngectomy. J. Clin. Otorhinolaryngol. Head Neck Surg..

[40] Teutsch S., Bas M., Bier H., Knopf A. (2016). Stomarezidive, eine klinisch-pathologische Betrachtung. Laryngo-Rhino-Otologie.

[41] Manfro G., Dias F.L., De Farias T.P. (2017). Tracheostomy Complications.

[42] Laccourreye O., Hans S., Borzog-Grayeli A., Maulard-Durdux C., Brasnu D., Housset M. (2000). Complications of postoperative radiation therapy after partial laryngectomy in supraglottic cancer: A long-term evaluation. Otolaryngol. Head Neck Surg..

[43] Lee J.H., Machtay M., Unger L.D., Weinstein G.S., Weber R.S., Chalian A.A., Rosenthal D.I. (1998). Prophylactic Gastrostomy Tubes in Patients Undergoing Intensive Irradiation for Cancer of the Head and Neck. Arch. Otolaryngol. Head Neck Surg..

[44] Chung E., Kim G., Cho B., Park H.S., Rho Y. (2016). Pattern of lymph node metastasis in hypopharyngeal squamous cell carcinoma and indications for level VI lymph node dissection. Head Neck.

[45] Pfister D.G., Laurie S.A., Weinstein G.S., Mendenhall W.M., Adelstein D.J., Ang K.K., Clayman G.L., Fisher S.G., Forastiere A.A., Harrison L.B. (2006). American Society of Clinical Oncology Clinical Practice Guideline for the Use of Larynx-Preservation Strategies in the Treatment of Laryngeal Cancer. J. Clin. Oncol..

[46] Robbins K.T., Clayman G., Levine P.A., Medina J., Sessions R., Shaha A., Som P., Wolf G.T. (2002). Neck dissection classification update: Revisions proposed by the American Head and Neck Society and the American Academy of Otolaryngology–Head and Neck Surgery. Arch. Otolaryngol. Head Neck Surg..

[47] Forastiere A.A., Zhang Q., Weber R.S., Maor M.H., Goepfert H., Pajak T.F., Morrison W.H., Glisson B.S., Trotti A., Ridge J.A. (2013). Long-Term Results of RTOG 91-11: A Comparison of Three Nonsurgical Treatment Strategies to Preserve the Larynx in Patients With Locally Advanced Larynx Cancer. J. Clin. Oncol..

[48] Langendijk J.A., Ferlito A., Takes R.P., Rodrigo J.P., Suárez C., Strojan P., Haigentz M., Rinaldo A., Tapia J.P.R. (2010). Postoperative strategies after primary surgery for squamous cell carcinoma of the head and neck. Oral Oncol..

[49] Le Tourneau C., Jung G.M., Borel C., Bronner G., Flesch H., Velten M. (2008). Prognostic factors of survival in head and neck cancer patients treated with surgery and postoperative radiation therapy. Acta Oto-Laryngol..

[50] Bernier J., Cooper J.S. (2005). Chemoradiation after Surgery for High-Risk Head and Neck Cancer Patients: How Strong Is the Evidence?. Oncologist.

[51] Group* DOVaLCS (1991). Induction chemotherapy plus radiation compared with surgery plus radiation in patients with advanced laryngeal cancer. N. Engl. J. Med..

[52] Blanchard P., Baujat B., Holostenco V., Bourredjem A., Baey C., Bourhis J., Pignon J.-P. (2011). Meta-analysis of chemotherapy in head and neck cancer (MACH-NC): A comprehensive analysis by tumour site. Radiother. Oncol..

[53] Weber R.S., Berkey B.A., Forastiere A., Cooper J., Maor M., Goepfert H., Morrison W., Glisson B., Trotti A., Ridge J.A. (2003). Outcome of salvage total laryngectomy following organ preservation therapy: The Radiation Therapy Oncology Group trial 91-11. Arch. Otolaryngol. Head Neck Surg..

[54] Budach V., Cho C.-H., Sedlmaier B., Wittlinger M., Iro H., Engenhart-Cabillic R., Hautmann M., Strutz J., Flentje M., Hueltenschmidt B. (2012). Five years’ results of the German ARO 04-01 trial of concurrent 72 Gy hyperfractionated accelerated radiation therapy (HART) plus once weekly cisplatinum/5-FU versus mitomycin C/5-FU in stage IV head and neck cancer. J. Clin. Oncol..

[55] Budach V., Stromberger C., Poettgen C., Baumann M., Budach W., Grabenbauer G., Marnitz S., Olze H., Wernecke K.-D., Ghadjar P. (2015). Hyperfractionated Accelerated Radiation Therapy (HART) of 70.6 Gy With Concurrent 5-FU/Mitomycin C Is Superior to HART of 77.6 Gy Alone in Locally Advanced Head and Neck Cancer: Long-term Results of the ARO 95-06 Randomized Phase III Trial. Int. J. Radiat. Oncol..

[56] Bauml J.M., Vinnakota R., Park Y.A., Bates S.E., Fojo T., Aggarwal C., Di Stefano J., Knepley C., Limaye S., Mamtani R. (2019). Cisplatin versus cetuximab with definitive concurrent radiotherapy for head and neck squamous cell carcinoma: An analysis of Veterans Health Affairs data. Cancer.

[57] Gillison M.L., Trotti A.M., Harris J., Eisbruch A., Harari P.M., Adelstein D.J., Jordan R.C.K., Zhao W., Sturgis E.M., Burtness B. (2019). Radiotherapy plus cetuximab or cisplatin in human papillomavirus-positive oropharyngeal cancer (NRG Oncology RTOG 1016): A randomised, multicentre, non-inferiority trial. Lancet.

[58] Xiang M., Holsinger F.C., Colevas A.D., Chen M.M., Beadle B.M., Beadle B.M. (2018). Survival of patients with head and neck cancer treated with definitive radiotherapy and concurrent cisplatin or concurrent cetuximab: A Surveillance, Epidemiology, and End Results-Medicare analysis. Cancer.

[59] Ferris R.L., Blumenschein G., Fayette J., Guigay J., Colevas A.D., Licitra L., Harrington K., Kasper S., Vokes E.E., Even C. (2016). Nivolumab for recurrent squamous-cell carcinoma of the head and neck. N. Engl. J. Med..

[60] Vermorken J.B., Mesia R., Rivera F., Remenar E., Kawecki A., Rottey S., Erfan J., Zabolotnyy D., Kienzer H.-R., Cupissol D. (2008). Platinum-Based Chemotherapy plus Cetuximab in Head and Neck Cancer. N. Engl. J. Med..

[61] Nuyts S., Lambrecht M., Duprez F., Daisne J.-F., Van Gestel D., Weyngaert D.V.D., Platteaux N., Geussens Y., Voordeckers M., Madani I. (2013). Reduction of the dose to the elective neck in head and neck squamous cell carcinoma, a randomized clinical trial using intensity modulated radiotherapy (IMRT). Dosimetrical analysis and effect on acute toxicity. Radiother. Oncol..

[62] Garden A.S., Harris J., Trotti A., Jones C.U., Carrascosa L., Cheng J.D., Spencer S.S., Forastiere A., Weber R.S., Ang K.K. (2008). Long-Term Results of Concomitant Boost Radiation Plus Concurrent Cisplatin for Advanced Head and Neck Carcinomas: A Phase II Trial of the Radiation Therapy Oncology Group (RTOG 99-14). Int. J. Radiat. Oncol..

[63] Machtay M., Moughan J., Trotti A., Garden A.S., Weber R.S., Cooper J.S., Forastiere A.A., Ang K.K. (2008). Factors Associated With Severe Late Toxicity After Concurrent Chemoradiation for Locally Advanced Head and Neck Cancer: An RTOG Analysis. J. Clin. Oncol..

[64] Haddad R., O’Neill A., Rabinowits G., Tishler R., Khuri F., Adkins D., Clark J., Sarlis N., Lorch J., Beitler J.J. (2013). Induction chemotherapy followed by concurrent chemoradiotherapy (sequential chemoradiotherapy) versus concurrent chemoradiotherapy alone in locally advanced head and neck cancer (PARADIGM): A randomised phase 3 trial. Lancet Oncol..

[65] Worden F.P., Moyer J., Lee J.S., Taylor J.M.G., Urba S.G., Eisbruch A., Teknos T.N., Chepeha D.B., Prince M.E., Hogikyan N. (2009). Chemoselection as a strategy for organ preservation in patients with T4 laryngeal squamous cell carcinoma with cartilage invasion. Laryngoscope.

[66] Budach W., Bölke E., Kammers K., Gerber P.A., Orth K., Gripp S., Matuschek C. (2016). Induction chemotherapy followed by concurrent radio-chemotherapy versus concurrent radio-chemotherapy alone as treatment of locally advanced squamous cell carcinoma of the head and neck (HNSCC): A meta-analysis of randomized trials. Radiother. Oncol..

[67] Dietz A., Wichmann G., Kuhnt T., Pfreundner L., Hagen R., Scheich M., Kölbl O., Hautmann M.G., Strutz J., Schreiber F. (2018). Induction chemotherapy (IC) followed by radiotherapy (RT) versus cetuximab plus IC and RT in advanced laryngeal/hypopharyngeal cancer resectable only by total laryngectomy—final results of the larynx organ preservation trial DeLOS-II. Ann. Oncol..

[68] Lefebvre J.-L., Ang K.K., Panel O.B.O.T.L.P.C. (2009). Larynx preservation clinical trial design: Key issues and recommendations-A consensus panel summary. Head Neck.

[69] Dziegielewski P.T., O’Connell D.A., Klein M., Fung C., Singh P., Mlynarek M.A., Fung D., Harris J.R., Seikaly H. (2012). Primary total laryngectomy versus organ preservation for T3/T4a laryngeal cancer: A population-based analysis of survival. J. Otolaryngol. Head Neck Surg..

[70] Timme D.W., Jonnalagadda S., Patel R., Rao K., Robbins K.T. (2015). Treatment selection for T3/T4a laryngeal cancer: Chemoradiation versus primary surgery. Ann. Otol. Rhinol. Laryngol..

[71] Sanabria A., Chaves A.L., Kowalski L.P., Wolf G.T., Saba N.F., Forastiere A.A., Beitler J.J., Nibu K.-I., Bradford C.R., Suárez C. (2017). Organ preservation with chemoradiation in advanced laryngeal cancer: The problem of generalizing results from randomized controlled trials. Auris Nasus Larynx.

[72] Grover S., Swisher-McClure S., Mitra N., Li J., Cohen R.B., Ahn P.H., Lukens J.N., Chalian A.A., Weinstein G.S., O’Malley B.W. (2015). Total Laryngectomy Versus Larynx Preservation for T4a Larynx Cancer: Patterns of Care and Survival Outcomes. Int. J. Radiat. Oncol..

[73] Eschmann S.-M., Paulsen F., Reimold M., Dittmann H., Welz S., Reischl G., Machulla H.-J., Bares R. (2005). Prognostic impact of hypoxia imaging with 18F-misonidazole PET in non-small cell lung cancer and head and neck cancer before radiotherapy. J. Nucl. Med..

[74] Mortensen L.S., Johansen J., Kallehauge J., Primdahl H., Busk M., Lassen P., Alsner J., Sørensen B.S., Toustrup K., Jakobsen S. (2012). FAZA PET/CT hypoxia imaging in patients with squamous cell carcinoma of the head and neck treated with radiotherapy: Results from the DAHANCA 24 trial. Radiother. Oncol..

[75] Hoffmann T.K. (2012). Systemic therapy strategies for head-neck carcinomas: Current status. GMS Curr. Top. Otorhinolaryngol. Head Neck Surg..

[76] Silverman D.A., Puram S.V., Rocco J.W., Old M.O., Kang S.Y. (2019). Salvage laryngectomy following organ-preservation therapy—An evidence-based review. Oral Oncol..

[77] Birkeland A.C., Rosko A.J., Issa M.R., Shuman A.G., Prince M.E., Wolf G.T., Bradford C.R., McHugh J.B., Brenner J.C., Spector M.E. (2016). Occult Nodal Disease Prevalence and Distribution in Recurrent Laryngeal Cancer Requiring Salvage Laryngectomy. Otolaryngol. Neck Surg..

[78] Hussain T., Kanaan O., Höing B., Dominas N., Lang S., Mattheis S. (2018). Die Rolle der elektiven Neck dissection bei Salvage Laryngektomie—Eine retrospektive Analyse. Laryngo-Rhino-Otologie.

[79] Rosko A., Birkeland A., Shuman A., Prince M., Bradford C., Wolf G., Worden F., Eisbruch A., Srinivasan A., Wong K.K. (2017). Positron emission tomography-CT prediction of occult nodal metastasis in recurrent laryngeal cancer. Head Neck.

[80] Al-Mamgani A., Tans L., Van Rooij P., Levendag P.C. (2012). A Single-Institutional Experience of 15 Years of Treating T3 Laryngeal Cancer With Primary Radiotherapy, With or Without Chemotherapy. Int. J. Radiat. Oncol..

[81] Rothmeier N., Hoffmann T.K., Lehnerdt G., Lang S., Mattheis S. (2013). Chirurgisches Management der persistierenden Speichelfistel nach Salvage-Laryngektomie. Laryngo-Rhino-Otologie.

[82] Goepfert R.P., Hutcheson K.A., Lewin J.S., Desai N.G., Zafereo M.E., Hessel A.C., Lewis C.M., Weber R.S., Gross N.D. (2017). Complications, hospital length of stay, and readmission after total laryngectomy. Cancer.

[83] Karsten R.T., Timmermans A.J., Cate J.T., Stuiver M.M., Brekel M.W.M.V.D. (2018). Direct complications and routine ICU admission after total laryngectomy. Acta Oto-Laryngologica.

[84] Helman S.N., Brant J.A., Kadakia S.K., Newman J.G., Cannady S.B., Chai R.L. (2018). Factors associated with complications in total laryngectomy without microvascular reconstruction. Head Neck.

[85] Hasan Z., Dwivedi R., Gunaratne D., Virk S., Palme C., Riffat F. (2017). Systematic review and meta-analysis of the complications of salvage total laryngectomy. Eur. J. Surg. Oncol. (EJSO).

[86] Hall F.T., O’Brien C.J., Clifford A.R., McNeil E.B., Bron L., Jackson M.A. (2003). Clinical outcome following total laryngectomy for cancer. ANZ J. Surg..

[87] Furuta Y., Homma A., Oridate N., Suzuki F., Hatakeyama H., Suzuki K., Nishioka T., Shirato H., Fukuda S. (2008). Surgical complications of salvage total laryngectomy following concurrent chemoradiotherapy. Int. J. Clin. Oncol..

[88] Upile T., Triaridis S., Kirkland P., Archer D., Searle A., Irving C., Evans P.R. (2005). The management of carotid artery rupture. Eur. Arch. Oto-Rhino-Laryngol..

[89] Grau C., Johansen L.V., Hansen H.S., Andersen E., Godballe C., Andersen L.J., Hald J., Møller H., Overgaard M., Bastholt L. (2003). Salvage laryngectomy and pharyngocutaneous fistulae after primary radiotherapy for head and neck cancer: A national survey from DAHANCA. Head Neck.

[90] Stankovic M., Milisavljevic D., Zivic M., Stojanov D., Stankovic P. (2015). Primary and salvage total laryngectomy. Influential factors, complications, and survival. J. BUON.

[91] Bratzler D.W., Dellinger E.P., Olsen K.M., Perl T.M., Auwaerter P.G., Bolon M.K., Fish D.N., Napolitano L.M., Sawyer R.G., Slain D. (2013). Clinical Practice Guidelines for Antimicrobial Prophylaxis in Surgery. Surg. Infect..

[92] Patel P.N., Jayawardena A.D.L., Walden R.L., Penn E.B., Francis D.O. (2018). Evidence-Based Use of Perioperative Antibiotics in Otolaryngology. Otolaryngol. Neck Surg..

[93] Dedivitis R.A., Aires F.T., Cernea C.R., Brandão L.G. (2015). Pharyngocutaneous fistula after total laryngectomy: Systematic review of risk factors. Head Neck.

[94] Formeister E.J., Alemi A.S., El-Sayed I., George J.R., Ha P., Knott P.D., Ryan W.R., Seth R., Tamplen M.L., Heaton C.M. (2018). Shorter interval between radiation therapy and salvage laryngopharyngeal surgery increases complication rates following microvascular free tissue transfer. Am. J. Otolaryngol..

[95] Miles B.A. (2017). Moving Toward Improved Outcomes in Salvage Laryngectomy. Ann. Surg. Oncol..

[96] Achim V., Bash J., Mowery A., Guimaraes A.R., Li R., Schindler J., Wax M., Andersen P., Clayburgh D. (2017). Prognostic Indication of Sarcopenia for Wound Complication After Total Laryngectomy. JAMA Otolaryngol. Neck Surg..

[97] Filimonov A., Brady J.S., Govindan A., Merchant A., Eloy J.A., Baredes S., Park R.C.W. (2017). Postoperative complications of total laryngectomy in diabetic patients. Laryngoscope.

[98] Gourin C.G., Stewart C.M., Frick K.D., Fakhry C., Pitman K.T., Eisele D.W., Austin J.M. (2019). Association of Hospital Volume With Laryngectomy Outcomes in Patients With Larynx Cancer. JAMA Otolaryngol. Neck Surg..

[99] Boenninghaus H.G., Lenarz T. (2012). Hals-Nasen-Ohren-Heilkunde.

[100] Eadie T.L., Bowker B.C. (2012). Coping and Quality of Life after Total Laryngectomy. Otolaryngol. Neck Surg..

[101] Slesina W., Rennert D., Weber A. (2014). Patientenbesuche im Krankenhaus durch Besuchsdienste von Krebs-Selbsthilfegruppen—Zur Prozess- und Ergebnisqualität. Das Gesundh..

[102] Lorenz K.J. (2014). Stimmrehabilitation nach Laryngektomie. HNO.

[103] Doescher J., Scheckenbach K., Angerstein W., Veit J.A., Schuler P.J., Laban S., Thierauf J., Theodoraki M.-N., Greve J., Hoffmann T.K. (2015). Evaluation of Customized Prosthesis for Irregularly Formed Tracheostoma After Laryngectomy. Ann. Otol. Rhinol. Laryngol..

[104] Hackenberg S., Kleinsasser N., Scherzad A., Kraus F., Hagen R. (2018). Microvascular reconstruction of the larynx following total laryngectomy. Laryngorhinootologie.

[105] Rosa V.M., Fores J.M.L., Da Silva E.P.F., Guterres E.O., Marcelino A., Nogueira P.C., Baia W.R.M., Kulcsar M.A.V. (2018). Interdisciplinary interventions in the perioperative rehabilitation of total laryngectomy: An integrative review. Clinics.

[106] Landis B.N., Giger R., Lacroix J.-S., Dulguerov P. (2003). Swimming, snorkeling, breathing, smelling, and motorcycling after total laryngectomy. Am. J. Med..

[107] Mallis A., Goumas P.D., Mastronikolis N.S., Panogeorgou T., Stathas T., Prodromaki K., Papadas T.A. (2011). Factors influencing quality of life after total laryngectomy: A study of 92 patients. Eur. Rev. Med. Pharmacol. Sci..

[108] Minovi A., Nowak C., Marek A. (2009). Quality of life bei Langzeitüberlebenden nach Laryngektomie. Laryngo-Rhino-Otologie.

[109] Patel R.S., Mohr T., Hartman C., Stach C., Sikora A.G., Zevallos J.P., Sandulache V.C. (2018). Tracheoesophageal Prosthesis Use Is Associated With Improved Overall Quality of Life in Veterans With Laryngeal Cancer. Ann. Otol. Rhinol. Laryngol..

[110] Hanna E., Sherman A., Cash D., Adams D., Vural E., Fan C.-Y., Suen J.Y. (2004). Quality of Life for Patients Following Total Laryngectomy vs Chemoradiation for Laryngeal Preservation. Arch. Otolaryngol. Head Neck Surg..

[111] Trivedi N.P., Swaminathan D.K., Thankappan K., Chatni S., Kuriakose M.A., Iyer S. (2008). Comparison of Quality of Life in Advanced Laryngeal Cancer Patients after Concurrent Chemoradiotherapy vs Total Laryngectomy. Otolaryngol. Neck Surg..

[112] LoTempio M.M., Wang K.H., Sadeghi A., DeLacure M.D., Juillard G.F., Wang M.B. (2005). Comparison of quality of life outcomes in laryngeal cancer patients following chemoradiation vs. total laryngectomy. Otolaryngol. Neck Surg..

